# Solution properties and aggregating structures for a fluorine-containing polymeric surfactant with a poly(ethylene oxide) macro-monomer

**DOI:** 10.1098/rsos.180610

**Published:** 2018-08-15

**Authors:** Xiaogang Wu, Chuanrong Zhong, Xiaofei Lian, Yan Yang

**Affiliations:** 1 State Key Laboratory of Oil and Gas Reservoir Geology and Exploitation, Chengdu University of Technology, Chengdu 610059, Sichuan, People's Republic of China; 2 College of Energy, Chengdu University of Technology, Chengdu 610059, Sichuan, People's Republic of China; 3 Down Hole Operation Sub-Company, Daqing Oilfield, Daqing 163000, Heilongjiang, People's Republic of China

**Keywords:** macro-monomer, fluorine-containing monomer, interfacial tension, ultraviolet analysis, transmission electron microscope

## Abstract

A polymeric surfactant (PFSA) was synthesized by the aqueous free-radical copolymerization using acrylamide, sodium 2-acrylamido-2-methylpropane sulfonate, allyl-capped octylphenoxy poly(ethylene oxide) (PEO) with the polymerization degree of 20 (AOP) and 1H,1H,2H,2H-perfluoro-1-decyl *p*-vinylbenzyl ether (VF). PFSA exhibited both the good surface and interfacial activities and the thickening behaviour. It could be used in enhanced oil recovery to increase both sweep and oil displacement efficiencies. The critical micelle concentration (CMC) of PFSA was 0.1 g l^−1^ in aqueous solution. The spherical micelles with the diameter of 100 nm were formed at CMC, and numerous compact worm-shaped micelles were observed above CMC. The interfacial tension was 0.027 mN m^−1^ for the 0.1 g l^−1^ PFSA solution containing 5 g l^−1^ NaCl and 0.209 g l^−1^ SDBS. The PFSA solutions still showed low interfacial tensions at high NaCl concentrations and temperatures, respectively, because of the incorporation of both VF and AOP containing long PEO.

## 1. Introduction

The molecular weights of polymeric surfactants are higher than 1000 g mol^−1^ and are traditionally 10^3^–10^4^ orders of magnitude. So, in comparison with the small-molecule surfactants, the polymeric surfactants can display the thickening property as well as the surface and interfacial activities. As polymeric surfactants are added to the injected aqueous phase in enhanced oil recovery (EOR) for the oil reservoirs with the medium-to-low permeability of 100–10 (10^−3^ µm^2^), the solution viscosities are increased, so the fingering of water through the more viscous crude oil can be avoided for some water-drive reservoirs with heterogeneity. In addition, the polymeric chains can flow through the very small pore throats. This does not give rise to the formation damage. As a result, not only the oil displacement efficiency but also the sweep efficiency is increased, and the oil recovery ratio is further enhanced [1,2]. Polymeric surfactants include natural and synthetic ones; the former are commonly hydrophobically modified cellulose [3–7], and the latter are linear and comb-like polymeric surfactants. However, the cellulose-based polymeric surfactants are not resistant to heat, their applied temperatures are lower than 60°C in oil reservoirs and their critical micelle concentrations (CMCs) are often much higher than 0.5 g l^−1^. The linear synthetic polymeric surfactants are mainly non-ionic poly(ethylene oxide)/poly(propylene oxide) (PEO/PPO) block copolymers [8–10]. Their surface tensions are low to 33 mN m^−1^ and are resistant to salts, especially multivalent metal cationic salts. However, their cloud points are lower than 55°C, at which the surfactants are precipitated from aqueous solutions. Thus, their used temperatures cannot be higher than 55°C in EOR.

The comb-like polymeric surfactants are ter-polymers or tetra-polymers in EOR [11,12]. They were conventionally synthesized from hydrophilic monomers such as acrylamide (AM) and/or acrylic acid, a hydrophobic monomer, e.g. styrene or dodecyl methacrylate [13,14], and a macro-monomer. The used macro-monomers are often alkyl PEO esters such as PEO esters of (meth)acrylic acid [15–18]. The average time when the oil-flooding agents stay in oil reservoirs is at least three months, so their molecular structures need to be stable for a long period. But the ester groups in these synthetic surfactants are easily hydrolysed at temperatures higher than 45°C. Thus, their anti-ageing properties are expected to be improved. In addition, their surface and interfacial activities should be enhanced to meet the needs of oil-flooding technique in EOR.

**Scheme 1.**
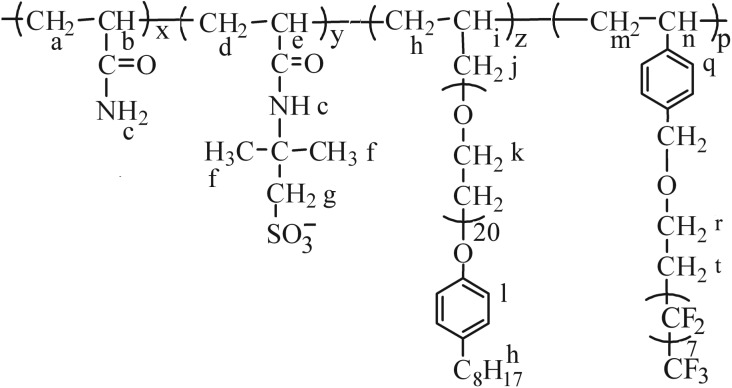
Schematic molecular structure of PFSA.

The applications of polymeric surfactants have been restricted in oil reservoirs for the aforementioned reasons. In this article, a fluorine-containing monomer and a macro-monomer with stable molecular structure were introduced to a polymeric surfactant to improve its heat resistance in oil reservoirs. In addition, the fluorine-containing groups are hydrophobic and oleophobic; therefore, the synthesized surfactant could make the crude oil on the rock surface exfoliate easily. This leads to the enhancement of the oil displacement efficiency in the medium-to-low-permeability oil reservoirs. So, a fluorine-containing monomer, 1H,1H,2H,2H-perfluoro-1-decyl *p*-vinylbenzyl ether (VF), was synthesized [19]. Then, a polymeric surfactant (PFSA, scheme 1) was copolymerized using AM, sodium 2-acrylamido-2-methylpropane sulfonate (NaAMPS), VF and a macro-monomer, allyl-capped octylphenoxy poly(ethylene oxide) with the polymerization degree of 20 (AOP) [20]. The AOP unit can expand the polymeric surfactant chain and make PFSA perform surface and interfacial behaviour, and the NaAMPS unit can improve the water solubility, salt resistance and heat resistance of PFSA. The effects of PFSA concentration, NaCl concentration and temperature on the surface and interfacial tensions of PFSA aqueous and brine solutions were investigated. The intermolecular interactions of PFSA in aqueous and brine solutions were studied with the ultraviolet (UV) analysis using pyrene as a probe. The micellar morphologies of PFSA in aqueous solution were observed using a transmission electron microscope (TEM).

## 2. Experimental section

### 2.1. Materials

AM was recrystallized twice from chloroform. 2-Acrylamido-2-methylpropane sulfonate (AMPS) was obtained from Lubrizol Co. (USA). 1H,1H,2H,2H-perfluoro-1-decanol (PFD) and *p*-vinylbenzyl chloride were purchased from Acros Organics Company. The AOP macro-monomer and the VF monomer were synthesized in our laboratory [19,20]. AM, NaCl, phosphotungstate, pyrene, sodium dodecyl benzene sulfonate (SDBS), kerosene and other reagents were analytically pure and were supplied by Chengdu KeLong Chemical Co. (China). The used water is distilled secondly.

### 2.2. Instruments and methods

Pyrene was used as a micropolarity-sensitive probe, and its concentrations in the solutions were determined by polymeric surfactant concentration and were (1–5) × 10^−5^ mol l^−1^. Then, the UV spectra were obtained with a UV-2450 spectrophotometer (Shimadzu Corporation, Japan). A solution of VF in CDCl_3_ and a solution of PFSA in D_2_O were measured with a 400 MHz Inova-400 instrument (Varian Company, USA) at room temperature. The FT-IR spectra were determined with a Nicolet-560 FT-IR spectrophotometer with a resolution capacity of 1 cm^−1^, and a scanning number was 32.

The carbon, hydrogen, nitrogen and sulfur contents of the polymer were measured with a Carlo Esra-1106 elemental analyser (Carlo Erba Co., Ltd, Italy). The molar percentage compositions of the PFSA polymers were calculated from the measured data using equations (2.1)–(2.8), where *A*, *E*, *F* and *P* are the moles of AM, AOP, VF and NaAMPS in 100 g of terpolymer, respectively. The coefficients are the numbers of carbon, hydrogen, nitrogen and sulfur in each monomer.

2.1 %C/12.01=3A+57E+19F+7P,

2.2 %N/14.01=1A+1P,

2.3 %H/1.01=5A+106E+13F+12P,

2.4 %S/32.01=1P,

2.5 mol%AM=100A/(A+E+F+P),

2.6 mol%AOP=100E/(A+E+F+P),

2.7 mol%VF=100F/(A+E+F+P)

2.8 andmol%NaAMPS=100P/(A+E+F+P).

The TEM images were obtained using an H-600 TEM (Hitachi Co., Ltd, Japan). The samples for TEM observations were prepared by placing 10 µl of polymeric surfactant solution on copper grids coated with thin films of formvar. Negative staining was done using an aqueous 2 wt% sodium phosphotungstate solution.

The intrinsic viscosities were measured with a 0.6 mm Ubbelohde capillary viscometer in a 1 mol l^−1^ sodium nitrate solution at (30.0 ± 0.1)°C. The apparent viscosities of polymeric surfactant aqueous and brine solutions were measured at 30°C and a shear rate of 7.34 s^−1^ with a DVIII R27112E viscometer (Brookfield Co., USA). The surface tensions were determined using the plate method with a Kruss-Kiost surface tensiometer (Kruss Co., Germany) at 30°C, and the interface tensions of polymeric surfactant solution/kerosene mixtures were measured with a TX500C spinning drop interface tensiometer (Bowing Industry Co., USA) at 30°C.

### 2.3. Synthesis of the PFSA polymeric surfactant

The PFSA polymeric surfactant was prepared by the aqueous free-radical copolymerization [20]. The copolymerization parameters of PFSA were optimized. AM (5.0 g, 0.07034 mol, 87.4 mol%) and AMPS (2.0016 g, 9.6577 × 10^−3^ mol, 12 mol%) were added to a 100 ml three-necked round-bottomed flask with a mechanical stirrer, a nitrogen inlet and an outlet. Then, 41.13 ml of distilled water was also added to the flask, and the mixture was stirred for 0.5 h till all solids were completely dissolved in water. NaOH was used to control the pH value of the reaction solution between 5 and 7. Sodium dodecyl sulfate (SDS) (1.0502 g) and AOP (0.2722 g, 2.4145 × 10^−4^ mol, 0.3 mol%) were added to the flask, and the solution was stirred for 20 min. Finally, VF (0.1401 g, 2.4145 × 10^−4^ mol, 0.3 mol%) was added to the solution. The solution was purged with N_2_ for 1 h to exclude oxygen gas and was then heated to 60°C in a tempering kettle under a nitrogen atmosphere. After that, 0.88 ml of 0.05 mol l^−1^ K_2_S_2_O_8_ solution was added to the solution to initiate the polymerization. The mass per cent composition of total monomer in water was 15%. The mixture was then polymerized for 36 h at 60°C to yield a transparent polymer gel. The polymer gel was dissolved in 700 ml of distilled water. And 1100 ml of isopropanol was then added to the aqueous solution with stirring to precipitate the PFSA polymeric surfactant. This surfactant was extracted with ethanol by the Soxhlet extractor for 2 days. And the surfactant was dried *in vacuo* at 50°C for 3 days.

The ^1^H-NMR shifts *δ* (ppm) for PFSA were as follows: H^a^, H^m^, 1.66; H^b^, 2.24; H^c^, 4.79; H^d^, 1.78; H^e^, 2.35; H^f^, H^i^, 1.49; H^g^, H^r^, 3.42; H^h^, 1.17–1.21; H^j^, H^n^, 3.21; H^k^, 3.63–3.69; H^l^, 6.99; H^q^, 7.72–7.78; H^t^, 2.06. FT-IR absorption peaks (cm^−1^) were: –N–H stretch, 3432.7; C=O stretch, 1670.1; –CH_3_, –CH_2_, –CH stretch, 2887.0, 2950.6, 2813.3; –CH_3_, –CH_2_, –CH bending, 1413.6, 1457.9, 1386.6; –CF_3_, –CF_2_ stretch, 1353.8, 1316.8; =C–H in phenyl stretch, 3218.7; C–O of PEO stretch, 1130.1; =C–O of phenol stretch, 1164.8; $-{\text{SO}}_{3}^{-}$: 1043.3, 648.0.

Under the same experimental conditions mentioned above, poly(AM-NaAMPS-AOP) (PAS) and poly(AM-NaAMPS-VF) (PFS) were synthesized and purified. The PFSA polymeric surfactant used in the following discussion was the above synthesized same sample.

## 3. Results and discussion

### 3.1. Effect of polymeric surfactant molecular structure on the surface tension

**Figure 1.**
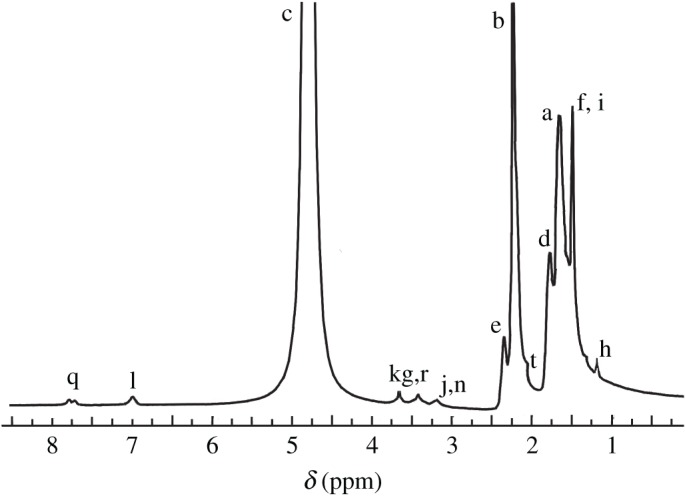
^1^H-NMR spectrum of PFSA.

Table 1 shows the polymer molar compositions of the synthesized PFSA, PAS and PFS. As shown in this table, the molar compositions of the NaAMPS, AOP and VF units in the three polymers were higher than the feed compositions, respectively. The AM and NaAMPS monomers are all dissolved in aqueous solution. But the random copolymerization tendency of NaAMPS is higher than that of AM because their reactivity ratios are *r*_AM_ = 0.98 and *r*_NaAMPS_ = 0.49. In addition, the local molar concentrations of AOP and VF solubilized in the SDS micelles should be, respectively, higher than their concentrations in aqueous solution, so the reactivity ratios of AOP and VF were also higher [20]. The weight-average molecular weights ($\bar{M\text{w}}$) of PFSA, PAS and PFS were, respectively, 8.65 × 10^4^ g mol^−1^, 14.1 × 10^4^ g mol^−1^ and 11.98 × 10^4^ g mol^−1^ according to the equation [*η*] = 3.15 × 10^−4^ $\bar{M\text{w}}\;$^0.7^ ([*η*]: intrinsic viscosity). The ^1^H-NMR spectrum is shown in figure 1 for PFSA. Figure 1 demonstrates the peaks ascribed to the groups of acrylamido, 2-methylpropane sulfonic ion $\left(-\text{C}{\left(\text{C}{\text{H}}_{3}\right)}_{2}\text{C}{\text{H}}_{2}{\text{SO}}_{3}^{-}\right)$, PEO chain, octylphenyl and benzyl for PFSA, and determines the polymerization of the AM, NaAMPS, VF and AOP monomers.

**Table 1.** Feed composition, elemental analysis and polymer composition of PFSA, PAS and PFS.

sample	feed composition M1 : M2 : M3 : M4^a^	elemental composition				polymer composition M1 : M2 : M3 : M4^b^	intrinsic viscosity (dl g^−1^)
		C (wt%)	N (wt%)	S (wt%)	H (wt%)		
PFSA	87.4 : 12.0 : 0.3 : 0.3	46.30	13.89	4.56	6.49	84.90 : 14.25 : 0.39 : 0.46	0.90
PAS	87.7 : 12.0 : 0.3 : 0	46.40	14.06	4.81	6.60	84.65 : 14.91 : 0.44 : 0	1.27
PFS	87.7 : 12.0 : 0 : 0.3	45.60	14.49	4.76	6.34	85.18 : 14.32 : 0 : 0.50	1.13

^a,b^M1, acrylamide; M2, NaAMPS; M3, AOP; M4, VF molar compositions.

**Figure 2.**
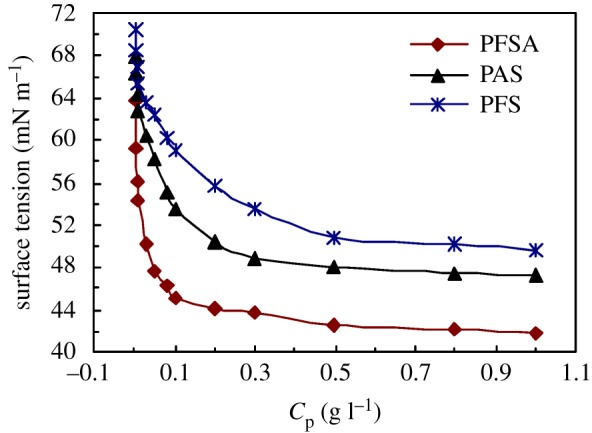
Effect of polymer concentration on surface tensions of aqueous solutions with different polymeric surfactants.

Figure 2 shows the effect of polymeric surfactant concentration on the surface tension (SFT) for three polymeric surfactants including PFSA, PAS and PFS in water. PFSA displayed the best surface activity in water. As the PFSA concentration was lower than 0.1 g l^−1^ in water, the surface tensions reduced sharply with an increase in polymeric surfactant concentration. The SFT was 45.10 mN m^−1^ in water at the PFSA concentration of 0.1 g l^−1^. Then, the surface tensions changed slightly above 0.1 g l^−1^. So, the CMC was 0.1 g l^−1^ for PFSA in water. By comparison, the surface tensions of the aqueous solutions were higher obviously for PAS than for PFSA at all polymer concentrations, CMC was 0.3 g l^−1^ and the SFT was 48.86 mN m^−1^ at CMC. Moreover, CMC of PFS was the highest for the three surfactants and was 0.5 g l^−1^, and the SFT was 50.87 mN m^−1^ at CMC. AOP is a macro-monomer with strong surface activities and contains a long PEO. So, the PAS chains were expanded in aqueous solution and PAS displayed comparatively low surface tensions because of the introduction of a small amount of AOP. Moreover, the VF monomer is hydrophobic and oleophobic, so the incorporation of this monomer could further decrease remarkably the surface tensions of polymer surfactant.

### 3.2. Effect of polymeric surfactant concentration on the apparent viscosity

**Figure 3.**
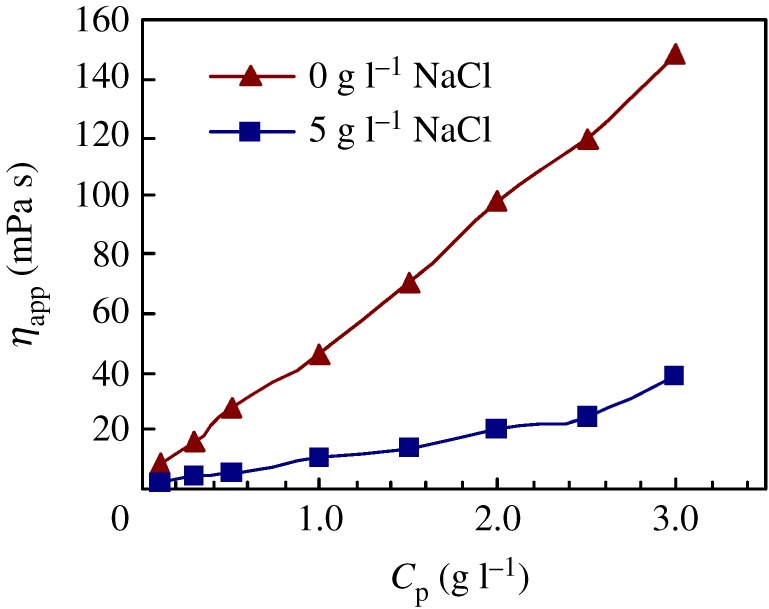
Effect of PFSA concentration on apparent viscosities in water and in 5 g l^−1^ NaCl.

Figure 3 shows the effect of polymeric surfactant concentration (*C*_p_) on apparent viscosities for PFSA in water and in a 5 g l^−1^ NaCl solution. As shown in figure 3, for PFSA in water and in the brine solution, the apparent viscosities increased remarkably with increasing polymer concentration, for example, the apparent viscosities of aqueous solutions were, respectively, 8 mPa s and 46 mPa s at the polymer concentrations of 0.1 g l^−1^ and 1.0 g l^−1^. With the addition of 5 g l^−1^ NaCl, the apparent viscosities became lower because of the electrostatic shielding effect, and the apparent viscosities decreased to, respectively, 3 mPa s and 11 mPa s in 0.1 g l^−1^ and 1.0 g l^−1^ PFSA. Thus, compared with small-molecule surfactants only presenting surface and interfacial activities, the PFSA surfactant exhibited an obvious thickening behaviour. As a result, as it is added to the aqueous phase in EOR, the fingering of water through the more viscous crude oil can be avoided, and the sweep efficiency of the injected water can be enhanced.

### 3.3. Effect of polymeric surfactant concentration on the surface and interfacial tensions

**Figure 4.**
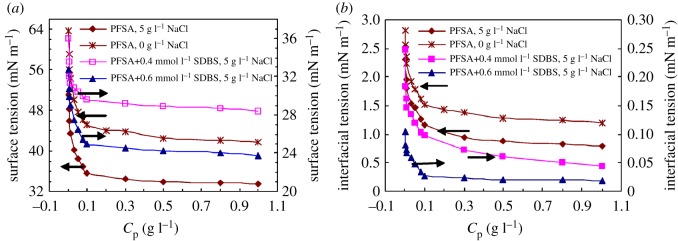
Effect of PFSA concentration on surface tensions (*a*) and interfacial tensions (*b*) in solutions.

**Figure 5.**
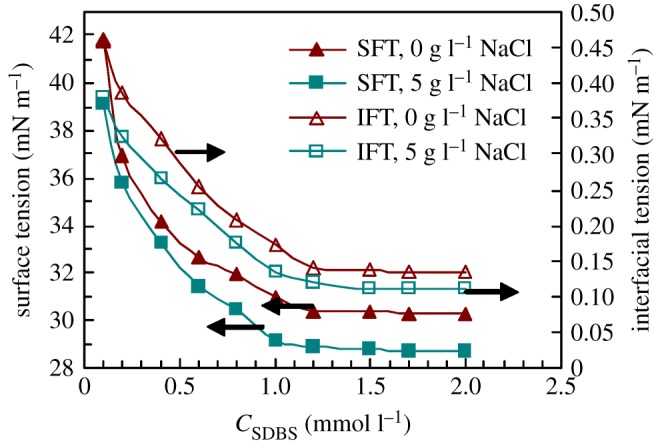
Effect of SDBS concentration on surface tensions and interfacial tensions in water and in 5 g l^−1^ NaCl.

Figure 4*a* shows the effect of polymeric surfactant concentration on surface tensions for PFSA in water and in a 5 g l^−1^ NaCl solution. And this figure also shows the effect of SDBS on surface tensions for PFSA in a 5 g l^−1^ NaCl solution. CMC was 0.1 g l^−1^ for PFSA in 5 g l^−1^ NaCl, and the SFT was 35.65 mN m^−1^ at CMC and was lower than that in water (45.10 mN m^−1^). The variation of SFT with PFSA concentration in the brine solution was similar to that in aqueous solution. For the present polymeric surfactants not containing fluorine, CMC was almost higher than 1.0 g l^−1^ and the surface tensions were commonly 37–55 mN m^−1^ at CMC [15,21–25]. Therefore, compared with the polymeric surfactants without C–F bonds reported in the literature, the surface activity of PFSA was stronger and its CMC in aqueous and brine solutions was much lower. In addition, the CMCs of PFSA in aqueous and brine solutions were also much lower than those of most small-molecule surfactants; for example, the CMC of SDBS in the solutions was 1.2 mmol l^−1^ (0.418 g l^−1^) (figure 5). This was attributed to the arrangement and intermolecular interactions of fluorine-containing groups and the octylphenyl groups on the surfaces of the PFSA solutions. Moreover, the introduction of the hydrophilic NaAMPS unit could make the PFSA chains expand in aqueous and brine solutions. This could facilitate the aggregation of the polymer chains on the surface of aqueous and brine solutions.

The surface tensions all decreased obviously for PFSA in 5 g l^−1^ NaCl with the addition of 0.4 and 0.6 mmol l^−1^ SDBS (shown in figure 4*a*). For example, by the addition of 0.6 mmol l^−1^ SDBS, the SFT decreased from primary 35.65 to 24.91 mN m^−1^ for the 0.1 g l^−1^ PFSA brine solution with 5 g l^−1^ NaCl. By comparison, the surface tensions of 0.6 mmol l^−1^ (0.209 g l^−1^) and 0.309 g l^−1^ (0.887 mmol l^−1^) SDBS brine solutions were, respectively, 31.45 and 29.76 mN m^−1^ (shown in figure 5); the SFT of 0.309 g l^−1^ PFSA brine solutions was 34.55 mN m^−1^. This indicated that more hydrophobic and oleophobic groups in PFSA side chains were arranged on the surface of the PFSA brine solution containing SDBS. Moreover, the mixed micelles were formed because of the bridging interaction of small amounts of SDBS. And they contained hydrophobic and oleophobic fluorine-containing groups, hydrophobic octylphenyl groups and dodecylphenyl groups in SDBS.

Figure 4*b* shows the interfacial tensions of PFSA solutions/kerosene as a function of polymeric surfactant concentration for PFSA in water and in the 5 g l^−1^ NaCl solution. As the polymeric surfactant concentrations were increased from 0.003 to 0.1 g l^−1^, the interfacial tensions decreased dramatically for the PFSA aqueous and brine solutions. Then, they decreased slightly at higher PFSA concentrations. The interfacial tensions were, respectively, 1.51 and 1.16 mN m^−1^ for the 0.1 g l^−1^ PFSA aqueous and brine solutions. This showed that the PFSA surfactant exhibited remarkable interface behaviour as well as thickening behaviour in the solutions at low polymer concentrations. Moreover, the interfacial tensions were decreased by the addition of salt because of electrostatic shielding from Na^+^ ions. The interfacial tensions are often 1.5–15 mN m^−1^ at CMC for the reported polymeric surfactants not containing fluorine [11,21,26]. Thus, the interfacial property of PFSA should be better by comparison. So far, the fluorine-containing surfactants applied in EOR are only small-molecule perfluoro-betaines and perfluoro-sulfobetaines [27]. And their interfacial tensions are higher than 1.5 mN m^−1^ at CMC [28]. This is not favourable for the emulsification of oil and the enhancement of oil displacement efficiency in oil reservoirs.

Figure 4*b* also shows that the interfacial tensions of the PFSA brine solution were reduced dramatically by the addition of 0.4 and 0.6 mmol l^−1^ SDBS. For example, as the PFSA concentration was 0.1 g l^−1^ in 5 g l^−1^ NaCl, the interfacial tensions were, respectively, reduced abruptly to 0.097 and 0.027 mN m^−1^. For the mixed surfactant brine solution with 0.1 g l^−1^ PFSA and 0.6 mmol l^−1^ (0.209 g l^−1^) SDBS, the total surfactant concentration was 0.309 g l^−1^. The interfacial tensions of the brine solutions were, respectively, 0.94 and 0.148 mN m^−1^ (figure 5) in 0.309 g l^−1^ PFSA and in 0.309 g l^−1^ (0.887 mmol l^−1^) SDBS. So, the interfacial tension (IFT) of the PFSA/SDBS mixed solution was much lower than those of the solutions containing a single surfactant. In contrast to the brine solution only containing PFSA, as the total concentration of PFSA and SDBS was lower than a mixed CMC (MCMC) for the PFSA/SDBS mixed brine solution, PFSA chains adsorbed more closely on the interface of the brine solution via the physical cross-link of SDBS. Consequently, the interfacial tension was decreased obviously. On the other hand, the PFSA chains and SDBS molecules aggregated with each other via the hydrophobic interactions of fluorine-containing groups, octylphenyl and dodecylphenyl groups in aqueous solutions above MCMC, so a large amount of mixed micelles were formed.

### 3.4. Effect of NaCl concentration on the surface and interfacial tensions

**Figure 6.**
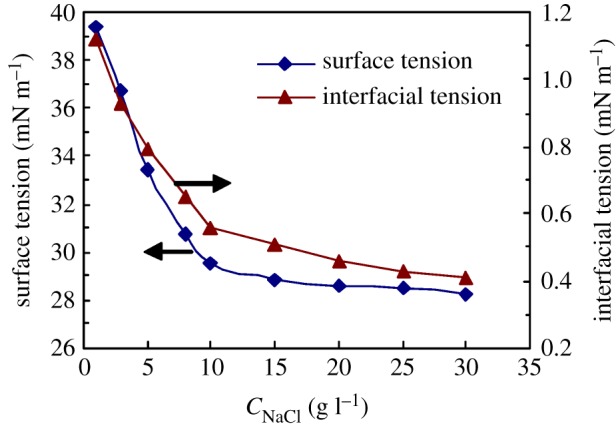
Effect of NaCl concentration on surface and interfacial tensions for 1.0 g l^−1^ PFSA brine solutions.

Figure 6 displays the effect of NaCl concentration on the surface and interfacial tensions for 1.0 g l^−1^ PFSA brine solutions. The PFSA polymeric surfactant presented good surface and interface activities in the brine solutions with 1–30 g l^−1^ NaCl. The surface and interfacial tensions reduced abruptly with an increase in the NaCl concentration from 1 to 10 g l^−1^ and then decreased slowly at higher NaCl concentrations. The surface and interfacial tensions were, respectively, 28.29 and 0.41 mN m^−1^ at a NaCl concentration of 30 g l^−1^. In comparison, for most small-molecule surfactants, the interfacial tensions decrease with increasing NaCl concentration because of the charge shielding effect at low NaCl concentrations, but increase remarkably at high NaCl concentrations. The charge repulsion of ions for a small-molecule surfactant is weakened by the addition of NaCl, so more surfactant molecules adsorb on the interface of oil/aqueous solution. However, as the NaCl concentration is generally higher than 10 g l^−1^, compact micelles aggregate together. Consequently, some surfactant precipitates from the brine solution and is then dissolved in the oil phase. So, the interface tension is increased. For PFSA, the strong electrostatic shielding from Na^+^ ions on $-{\text{SO}}_{3}^{-}$ groups was produced by the addition of salt at the NaCl concentrations lower than 10 g l^−1^ NaCl. And the PFSA chains arranged more closely on the interfaces of brine solutions. This resulted in an obvious decrease in IFT. At higher NaCl concentrations, Na^+^ ions could complex with the C–O bonds in the PEO side chains. As a result, the electrostatic shielding in the comb-like PFSA chains was weakened. Moreover, both the fluorine-containing groups with phenyl groups at their beginning and the long side chains with phenyl groups at their ends could enhance the rigidity of the polymer backbones. Consequently, the PFSA polymeric surfactant was still easily dissolved in the brine solutions at high NaCl concentrations.

### 3.5. Effect of temperature on the surface and interfacial tensions

**Figure 7.**
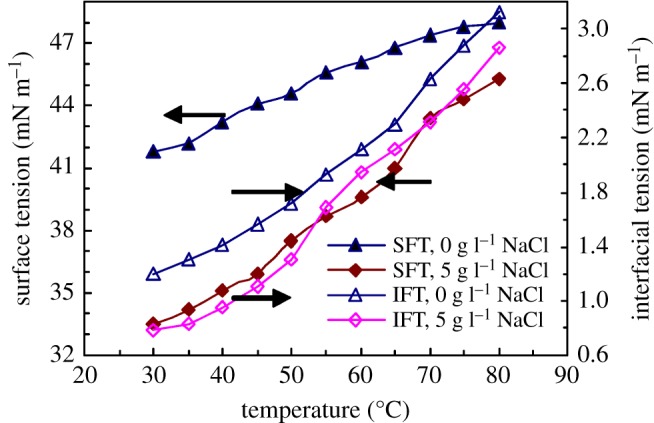
Effect of temperature on SFT and IFT for 1.0 g l^−1^ PFSA solutions.

Figure 7 shows the plots of SFT and IFT versus temperature for 1.0 g l^−1^ PFSA in water and in a 5 g l^−1^ NaCl solution. As the temperature was increased, for PFSA in the unsalted and brine solutions, the surface and interfacial tensions increased, and the surface and interface activities of PFSA were weakened. But at a high temperature of 80°C in water and in the brine solution, the surface tensions still remained, respectively, 48.03 and 45.27 mN m^−1^, and the interfacial tensions were 3.12 and 2.87 mN m^−1^, respectively. All of these data were much lower than ones of most reported polymeric surfactants at the same temperature. This showed that PFSA still exhibited comparatively good surface and interfacial activities at high temperatures. As the temperature was increased, on the one hand, the PFSA polymeric chains moved faster, and the aggregation of fluorine-containing groups and octylphenyl groups on the surface or interface of the brine solution was weakened obviously. On the other hand, some hydrogen bonds formed by the C–O bonds in PEO side chains and the water molecules were destroyed, resulting in a decrease in the hydrophilicity of PEO chains; accordingly, the intermolecular interactions of the hydrophobic groups on the surface or interface of the brine solution were reinforced. Thus, the surface and interfacial tensions of the PFSA brine solution were still comparatively low at high temperatures.

### 3.6. Effect of PFSA concentration on hydrophobic micro-domains

Pyrene is hardly dissolved in water, and its solubility is merely 1.0 × 10^−7^ mol l^−1^. However, it is preferentially solubilized into hydrophobic micro-domains, and an ultraviolet absorbance peak appears at 273 nm or so for its UV spectrum in aqueous solutions. As the sizes, compact degree and number of the hydrophobic micro-domains located by pyrene change very slightly, the absorbance value varies obviously. So, pyrene is used conventionally to probe the formation and non-polarity of micelles in water and brine solutions [29].

**Figure 8.**
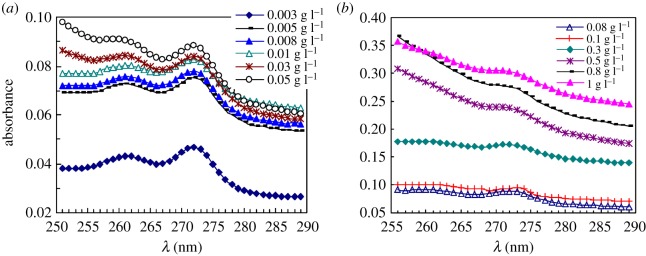
Ultraviolet spectra of pyrene in unsalted 0.003–0.008 g l^−1^ (*a*) and 0.01–1.0 g l^−1^ (*b*) PFSA solutions.

**Figure 9.**
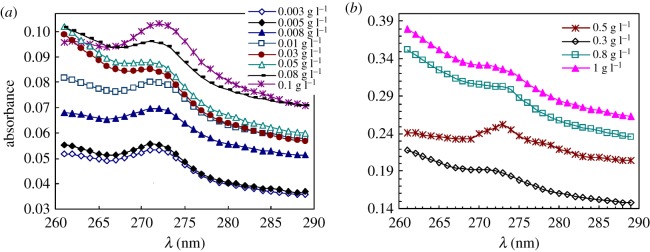
Ultraviolet spectra of pyrene in the 0.003–0.008 g l^−1^ (*a*) and 0.01–1.0 g l^−1^ (*b*) PFSA brine solutions. NaCl concentration: 5 g l^−1^ NaCl.

**Figure 10.**
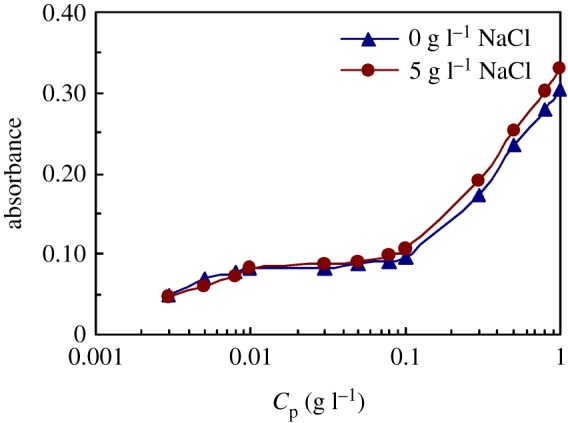
Effect of PFSA concentration on absorbance values for pyrene in unsalted and 5 g l^−1^ NaCl solutions.

Figure 8*a*,*b* displays the UV spectra of pyrene for PFSA in extremely dilute and dilute aqueous solutions at 30°C. Figure 9*a*,*b* shows the UV spectra of pyrene for the same PFSA sample in 5 g l^−1^ NaCl solutions at 30°C. The influence of PFSA concentration on the absorbance (*A*) value at approximately 273 nm is shown in figure 10 for these solutions. The *A* value of pyrene in pure water is 0.0430. The *A* values were only 0.0489–0.0828 for the aqueous 0.003–0.01 g l^−1^ PFSA solution. An inflection point appeared at the polymeric concentration of 0.03 g l^−1^, and the *A* value was 0.0830. The *A* value increased slowly from 0.0830 to 0.0962 with an increase in the PFSA concentration from 0.03 to 0.1 g l^−1^, and then rose abruptly above 0.1 g l^−1^. Finally, as the PFSA concentration was 1.0 g l^−1^, the *A* value was up to 0.3046. The *A* data demonstrated that the CMC was 0.1 g l^−1^ in water. The *A* values indicated that all PFSA macromolecular chains should be mono-molecular in aqueous solutions below 0.03 g l^−1^. As the PFSA concentration was increased from 0.03 to 0.1 g l^−1^, the number of octylphenyl groups and fluorine-containing groups arranged on the surfaces or interfaces of these aqueous solutions increased, and their intermolecular interactions were strengthened. Consequently, the SFT or IFT decreased dramatically. In addition, in these aqueous solutions, some aggregates were formed by the self-assembling of polymeric surfactant chains, and the number of aggregates increased with increasing surfactant concentration. As the PFSA concentration was higher than 0.1 g l^−1^, the octylphenyl groups and fluorine-containing groups on the surface or interface of the solutions were almost constant. Thus, the SFT or IFT changed very slowly. Moreover, the increased PFSA chains aggregated with each other via the intermolecular interactions to form more micelles with an increase in the PFSA concentration. As a result, a large amount of pyrene molecules were dissolved in these micelles with hydrophobic micro-domains, resulting in the high *A* values.

For the 5 g l^−1^ NaCl solutions with the same PFSA concentrations of 0.003–0.008 g l^−1^, the *A* values were lower very slightly than those in water, for example, at the PFSA concentration of 0.008 g l^−1^, the *A* value in the brine solution was 0.0727, which was almost equal to that (0.0779) in water. However, for the same 0.01–1.0 g l^−1^ PFSA solutions, the *A* values were increased very slightly by the addition of NaCl. These data indicated that almost all PFSA chains are also mono-molecular in the extremely dilute brine solutions and that the hydrophobic microstructures were not formed. In addition, this also indicated that the PFSA chains were comparatively expanded in the brine solutions as well as in unsalted aqueous solution at all surfactant concentrations. The expanded conformations of the surfactant chains in the solutions were due to the simultaneous incorporation of both the bulky fluorine-containing groups and the long PEO chains all containing the phenyl groups. For the same 0.01–1.0 g l^−1^ PFSA solutions, the arrangement of the octylphenyl groups and the fluorine-containing groups with the phenyl groups on their surface or interface and the hydrophobic micro-domains of micelles in the solutions were made more compact by the addition of NaCl. This was attributed to the charge shielding of the Na^+^ ions and the complexation of the Na^+^ ions with the C–O bonds in the PEO side chains. As a result, the surface and interfacial tensions of these brine solutions were remarkably lower than those of the unsalted solutions.

### 3.7. Effect of NaCl concentration on hydrophobic micro-domains

**Figure 11.**
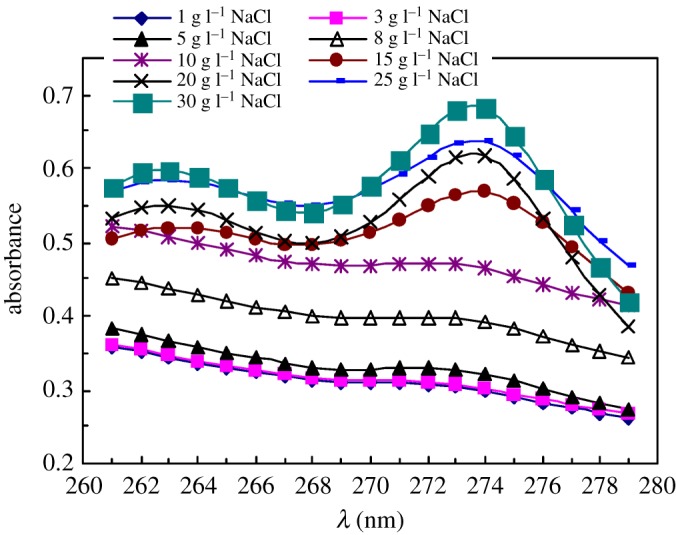
UV spectra of pyrene at different NaCl concentrations for 1.0 g l^−1^ PFSA brine solutions at 30°C.

**Figure 12.**
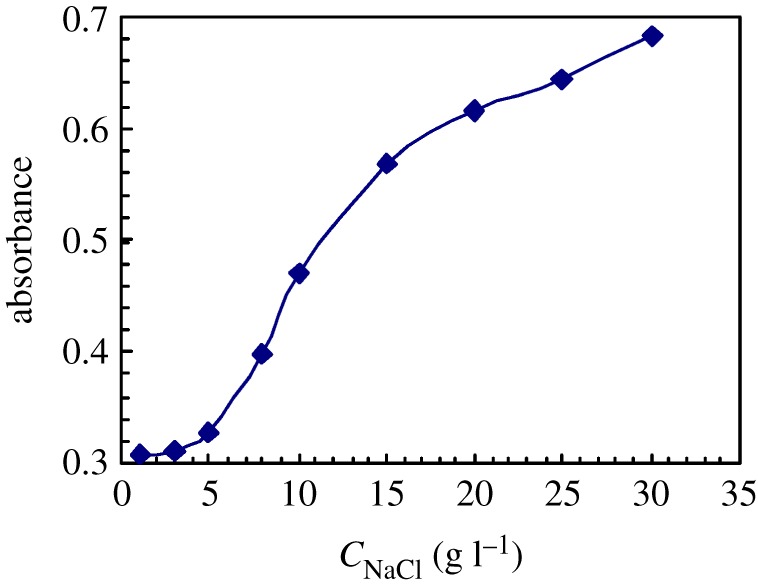
Effect of NaCl concentration on absorbance values for pyrene in 1.0 g l^−1^ PFSA brine solutions at 30°C.

Figure 11 shows the UV spectra of pyrene at different NaCl concentrations for 1.0 g l^−1^ PFSA brine solutions at 30°C. Figure 12 displays the variation of the absorbance (*A*) value at approximately 273 nm with NaCl concentration. As the NaCl concentration was increased below 5 g l^−1^, the *A* value increased slightly and then it rose abruptly above 5 g l^−1^. Finally, as the PFSA concentration was higher than 15 g l^−1^, the *A* value increased gradually. And as the PFSA concentration was increased to 30 g l^−1^, the *A* value was 0.6823. The charge shielding from Na^+^ ions on repulsive interactions of $-{\text{SO}}_{3}^{-}$ groups along PFSA chains became stronger with an increase in NaCl concentration. This resulted in the sharp reduction in the surface or interfacial tensions of the brine solution. But as the NaCl concentration was further increased from 15 to 30 g l^−1^, the electrostatic shielding from Na^+^ on $-{\text{SO}}_{3}^{-}$ groups was weakened by the complexation of some Na^+^ ions with the C–O bonds in the PEO side chains. Accordingly, the intermolecular interactions of the fluorine-containing groups and the octylphenyl groups were reinforced more slowly on the surfaces or interfaces of the brine solutions and in the micelles of brine solutions, so the surfaces or interfacial tensions of the brine solutions decreased slowly.

### 3.8. Effect of temperature on hydrophobic micro-domains

**Figure 13.**
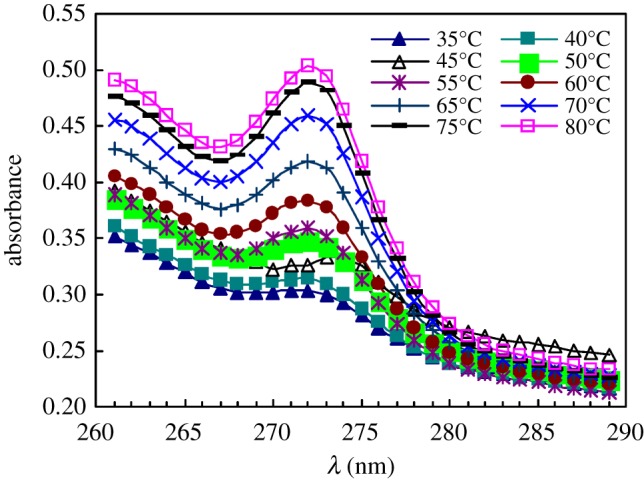
UV spectra of pyrene at different temperatures for 1.0 g l^−1^ PFSA brine solutions with 5.0 g l^−1^ NaCl.

**Figure 14.**
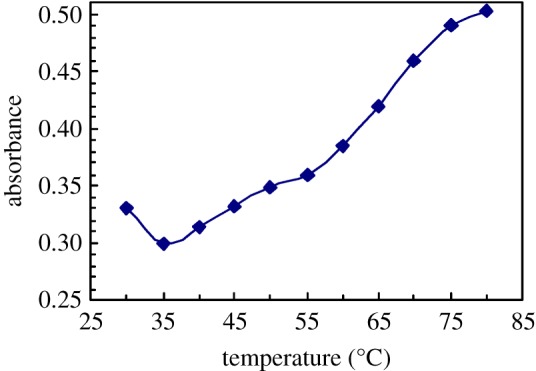
Effect of temperature on absorbance values for pyrene in 1.0 g l^−1^ PFSA brine solutions with 5.0 g l^−1^ NaCl.

Figure 13 shows the UV spectra of pyrene at different temperatures for 1.0 g l^−1^ PFSA brine solutions with 5.0 g l^−1^ NaCl. The absorbance (*A*) value at approximately 273 nm as a function of temperature is shown in figure 14. As the temperature was increased from 30 to 35°C, the *A* value decreased from 0.3292 to 0.2997. Then, the *A* value increased all through with increasing temperature, and the *A* value was 0.5031 at 80°C. These results suggested that faster movement of polymer chains could interfere with the aggregation of fluorine-containing groups and octylphenyl groups on the surface or interface of the brine solution and in the brine solution with an increase from 30 to 35°C. As the temperature was further increased, the arrangement of the PFSA polymeric chains on the surface or interface of the brine solution became looser, and consequently, the SFT or IFT of the brine solution increased. However, with an increase in temperature, more polymeric surfactant chains moved from the surface or interface into the solution and more micelles were formed in the brine solution. In addition, some hydrogen bonds were formed by the C–O bonds in PEO side chains and the water molecules were disrupted with increasing temperature. As a result, the hydrophilicity of PEO chains was decreased. So, the van der Waals intermolecular interactions of fluorine-containing groups and octylphenyl groups were reinforced, and hydrophobic micro-domains became more compact, where more pyrene was dissolved.

### 3.9. TEM morphologies of PFSA in aqueous solutions

**Figure 15.**
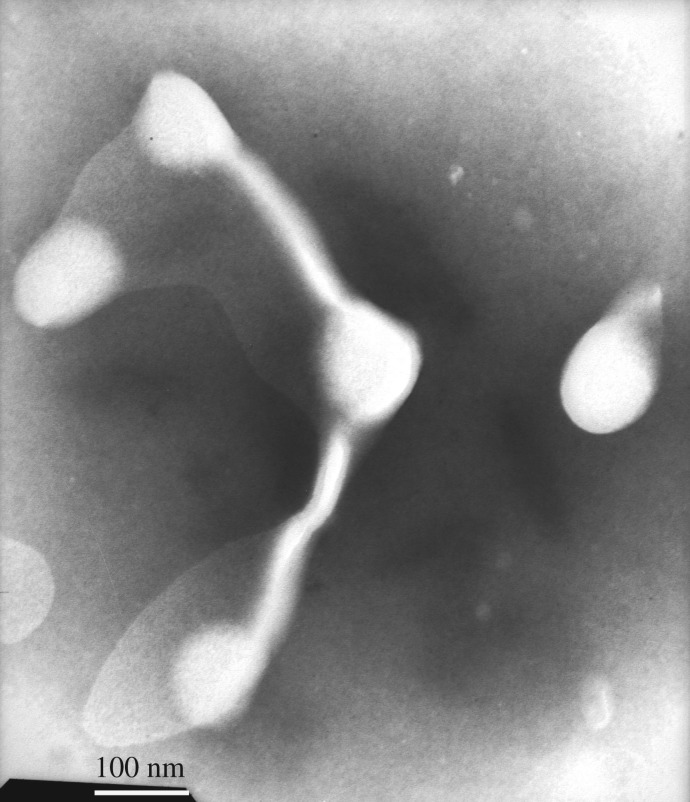
TEM image of an aqueous 0.1 g l^−1^ PFSA solution.

**Figure 16.**
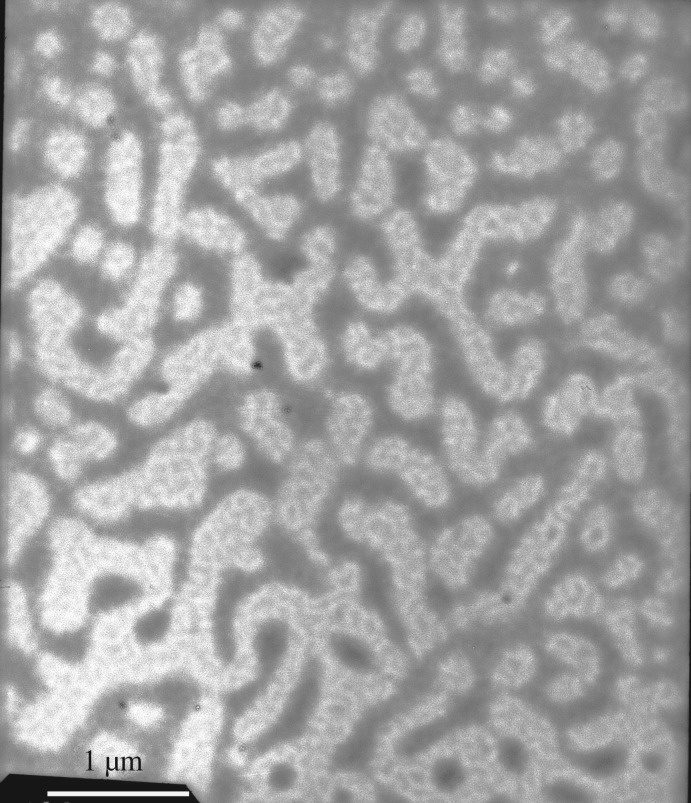
TEM image of an aqueous 0.3 g l^−1^ PFSA solution.

**Figure 17.**
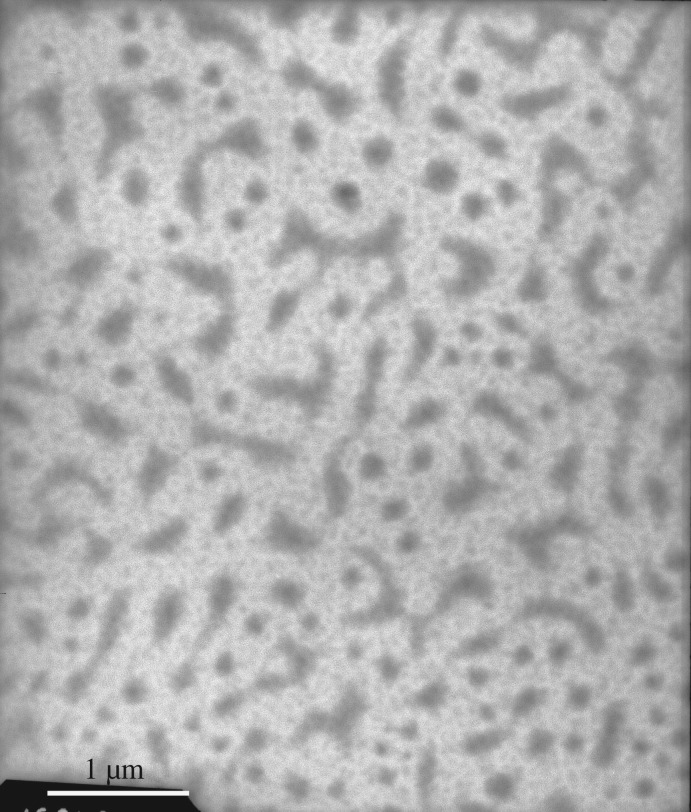
TEM image of an aqueous 0.4 g l^−1^ PFSA solution.

**Figure 18.**
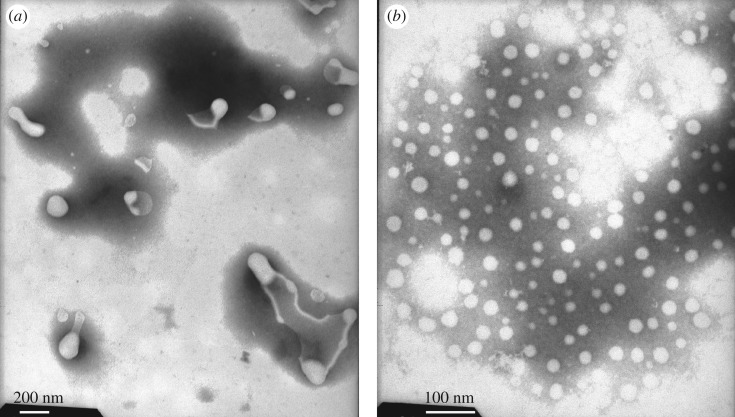
TEM image of an aqueous 0.5 g l^−1^ PFSA solution.

The PFSA solution samples were negatively stained. The electronic staining agent was water-soluble sodium phosphotungstate, and the black backgrounds were formed, i.e. the sites of negative stain were the black sections in TEM images. After the aqueous PFSA solutions on copper grids were dried under extremely high vacuum, the formed supra-molecular structures were white sections in TEM images. In contrast to the aggregated structures of the PFSA surfactant, the electronic density of sodium phosphotungstate was much higher. Consequently, the electron beams did not penetrate it and the black sections were formed in the images. Figure 15 shows the TEM image of an aqueous 0.1 g l^−1^ PFSA solution. As shown in figure 15, some spherical and expanded chain-like micelles were observed. The chain-like micelles should be formed by several polymeric surfactant chains. The diameters of the spherical micelles were 100 nm, and the diameters and lengths of the chain-like micelles were, respectively, 30 nm and 200–230 nm. This indicated that these polymer chains should contain more hydrophilic $-{\text{SO}}_{3}^{-}$ groups and fewer VE and AOP units compared with the spherical micelles. As the PFSA concentration was increased to 0.3 g l^−1^, being higher than CMC of PFSA, spherical micelles and chain-like micelles aggregated with each other to form a large number of worm-like structures (figure 16), and the intermolecular interactions of the hydrophobic and oleophobic groups in the solution and on the surface or interface of the solution were strengthened. So, the *A* value increased obviously from 0.0962 in 0.1 g l^−1^ PFSA to 0.1721 at 0.3 g l^−1^ for the UV spectra of pyrene, and the surface or interfacial tensions were low. As the PFSA concentration was further increased to 0.4 g l^−1^, the worm-like structures crossed each other to form continuous network structures (figure 17). The TEM images of an aqueous 0.5 g l^−1^ PFSA are shown in figure 18*a*,*b*, and the two images were, respectively, obtained from two different observed points of the same sample. As shown in figure 18*a*, the film-shaped structures were found because of the aggregation of a very large number of PFSA chains and spherical micelles. In addition, a small number of spherical and expanded chain-like micelles were also displayed in figure 18*a*. Some spherical micelles (diameter: 12–30 nm) were also observed in figure 18*b*, and the sizes of major particles were 30 nm. This suggested that the structures of these micelles became more compact because of the stronger intermolecular interactions compared with the spherical micelles (diameter: 100 nm) in 0.1 g l^−1^ PFSA. Therefore, for 0.5 g l^−1^ PFSA, the amount of pyrene dissolved in these hydrophobic micro-domains was also large and the *A* value increased from 0.0962 in 0.1 g l^−1^ to 0.2346.

## 4. Conclusion

The simultaneous incorporation of the AOP macro-monomer with surface activity and the fluorine-containing VF monomer made the PFSA polymeric surfactant exhibit the good surface and interfacial activities and the viscous behaviour. All PFSA chains were mono-molecular in aqueous solutions as well as adsorbed on the surface or interface of the solutions at extremely low surfactant concentrations, i.e. below 0.03 g l^−1^. The CMCs were very low and only 0.1 g l^−1^ for PFSA in aqueous solution and in 5 g l^−1^ NaCl, and the diameters of spherical micelles were 100 nm in aqueous solution. The IFT was 1.16 mN m^−1^ in brine solution at CMC and was decreased sharply to 10^−2^ magnitude order with the addition of a small amount of SDBS. The intermolecular interactions of octylphenyl groups and fluorine-containing groups in PFSA chains were reinforced with increasing polymer concentration, and micellar structures were remarkably increased above CMC. The complexation of Na^+^ ions with the C–O bonds in the PEO side chains and the rigidity of the PFSA chains resulted in low surface and interfacial tensions even at high NaCl concentrations for PFSA. Hydrophobic micro-domains became more compact in brine solutions with an increase in NaCl concentration. Although the IFT increased with increasing temperature, the IFT was still lower than 3.0 mN m^−1^ in 5 g l^−1^ NaCl at 80°C and micellar structures became more compact because of the destruction of some hydrogen bonds formed by the C–O bonds in PEO side chains and the water molecules.

## Data accessibility

Our data are deposited at Dryad: http://dx.doi.org/10.5061/dryad.bg8s00p [30].
